# One dollar incentive improves tuberculosis treatment outcomes in programmatic settings in rural Uganda

**DOI:** 10.1038/s41598-021-98770-7

**Published:** 2021-09-29

**Authors:** Joseph Baruch Baluku, Bridget Nakazibwe, Bright Twinomugisha, Rebecca Najjuuko, Nyirazihawe Isabella, Sylvia Nassozi, Sharon Namiiro, Winceslaus Katagira, Dathan Mirembe Byonanebye, Christine Sekaggya-Wiltshire, Joseph Muchiri, Elizabeth Ndungu, Godwin Anguzu, Harriet Mayanja-Kizza, Irene Andia-Biraro

**Affiliations:** 1 Department of Internal Medicine, Mulago National Referral Hospital, Kampala, Uganda; 2 Makerere University Lung Institute, Kampala, Uganda; 3 Directorate of Programs, Mildmay Uganda, Wakiso, PO Box 26343, Kampala, Uganda; 4 Masaka Regional Referral Hospital, Masaka, Uganda; 5 School of Public Health, Makerere University College of Health Sciences, Kampala, Uganda; 6 Department of Community Health, School of Public health, Mount Kenya University, Thika, Kenya; 7 Department of Community Health Nursing, Mount Kenya University, Thika, Kenya; 8 Makerere University Infectious Disease Institute, Kampala, Uganda; 9 MRC/UVRI & LSHTM Uganda Research Unit, Entebbe, Uganda; 10 Department of Clinical Research, Faculty of Infectious and Tropical Disease (ITD), London School of Hygiene and Tropical Medicine, London, UK

**Keywords:** Infectious diseases, Respiratory tract diseases, Health services

## Abstract

The study aim was to determine the association of a one United States dollar (USD) dollar incentive and tuberculosis (TB) treatment outcomes among people with TB receiving treatment at a rural hospital in Uganda under programmatic settings. We conducted a quasi-experiment in which people with TB were randomised (1:1 ratio) to receive either a one USD incentive at months 0, 2, 5 and 6 (Dollar arm) or routine care (Routine arm). A second control group (Retrospective controls) consisted of participants who had a treatment outcome in the preceding 6 months. Treatment outcomes were compared between the intervention and control groups using Pearson’s chi-square and Fisher’s exact tests. The association between the incentive and treatment outcomes was determined using Poisson regression analysis with robust variances. Between November 2018 and October 2019, we enrolled 180 participants (60 in the Dollar arm and 120 in the Control group). TB cure (33.3% vs. 20.8%, *p* = 0.068) and treatment success (70.0% vs. 59.2% *p *= 0.156) were higher in the Dollar arm than the Control group, while loss-to-follow-up was lower in the Dollar arm (10.0% vs. 20.8% *p* = 0.070). Participants in the Dollar arm were more likely to be cured (adjusted incidence rate ratio (aIRR): 1.59, 95% CI 1.04–2.44, *p* = 0.032) and less likely to be lost to follow-up (aIRR: 0.44, 95% CI 0.20–0.96, *p* = 0.040). A one-dollar incentive was associated with higher TB cure and lower loss-to-follow-up among people with TB in rural Uganda.

## Introduction

Globally, tuberculosis (TB) was the leading cause of death from an infectious agent in 2019^1^. In sub-Saharan Africa, only 76% of people with TB achieve treatment success despite a global target of 90%^2^. Within low-income countries (LICs), rural settings report disproportionately higher TB mortality and treatment attrition than urban settings, and this could be attributed to low socio-economic status among rural dwellers^3,4^. A study conducted in Uganda among people with TB co-infected with HIV found that participants attending urban facilities were four-fold more likely to achieve treatment success than those at rural facilities^5^. However, the socioeconomic attributes of the participants were not evaluated. Nonetheless, low socio-economic status is widely recognised as a risk factor for TB infection, TB disease, delayed TB diagnosis and treatment, and treatment attrition^6^. Therefore, the World Health Organisation (WHO) recognises social protection as a key component of the pillars of TB control and elimination in the End-TB Strategy^7^. Social protection strategies such as cash transfer programs, education, unemployment insurance, food provision and transport incentives have been shown to reduce the incidence of TB and improve treatment adherence, TB treatment success and cure^8–10^. Specifically, a recent meta-analysis has shown that cash incentives given to patients during TB treatment are associated with a 1.8-fold increase in favourable TB treatment outcome^11^. Moreover, cash incentives are not only more effective but are also preferred to non-monetary incentives by people with TB^12,13^. The amount of cash incentives used in studies to improve TB outcomes has been reported to range between 193 and 858 international dollars (a hypothetical currency that estimates how much goods or services would cost in a given country in comparison to similar goods and services in the United States in a given year)^11^. Unfortunately, these cash incentive amounts are too high for LICs to integrate into routine programmatic TB care without external funding. It is therefore important to determine whether a small “affordable” cash incentive would improve TB treatment outcomes in the most vulnerable populations, such as rural dwellers.

In Uganda, a TB/HIV high burdened country, 53% of households of people with TB experience catastrophic costs during TB treatment of which 42% are non-medical costs^14^. Notwithstanding, only 4% of these patients receive any form of social protection^15^. In rural Uganda, people with TB spend 15–100% of their household income on a single visit while seeking TB services from health facilities^16^. Therefore, cash incentives are likely to be effective in rural areas although there is a dearth of evidence from these settings. A study in rural Uganda has estimated that the least an individual with TB symptoms spends during a clinic visit at a government facility is approximately one USD^16^. The aim of this study was to determine the association of a one-dollar incentive and TB treatment outcomes among people with TB receiving treatment at a rural hospital in Uganda under programmatic settings. The study therefore provides preliminary evidence for a possible role of a low-cost incentive in improving TB outcomes in rural settings.

## Materials and methods

### Study objectives

The primary objectives were to determine the association of a one-dollar incentive with (i) the overall treatment success rate and (ii) loss-to-follow-up rate among people with TB receiving treatment at a rural hospital in Uganda under programmatic settings. The secondary objectives were to determine the association of a one-dollar incentive with (i) death and (ii) treatment failure among people with TB receiving treatment at a rural hospital in Uganda under programmatic settings.

### Study design and setting

We conducted a quasi-experiment that consisted of a prospective two-arm pragmatic randomised open-label non-placebo-controlled trial and a retrospective cohort at Masaka Regional Referral Hospital (MRRH) in rural central Uganda. The retrospective cohort consisted of a second control group of participants that had a documented TB treatment outcome in the immediate period (6 months) preceding the trial. MRRH is a tertiary regional hospital with a 330-bed capacity and annual admission of about 23,450 patients. The facility is located in Masaka district, 120 km from Kampala (the capital city of Uganda). MRRH serves as a regional referral center for eight rural districts in central Uganda with the respective percentage of rural residents: Masaka (65%), Rakai (93%), Lyantonde (85%), Lwengo (84%), Ssembabule (93%), Bukomansimbi (92%), Kalungu (82%) and Kalangala (91%) districts^17^. The hospital has a regional treatment centre for drug-sensitive and drug-resistant TB (DS- and DR-TB, respectively). On average, 21 People with TB are initiated on TB therapy at the TB diagnostic and treatment unit of the hospital per month.

### Study population and randomisation

Eligible participants were adults (≥ 18 years) initiating a 6-months TB treatment regimen at the study site. We excluded participants who were already on TB therapy at presentation to the study site. Further, we excluded those with DR-TB and those with TB of the bone or central nervous system because their treatment duration conventionally exceeds six months. Additionally, participants with DR-TB routinely receive non-monetary enablers and cash transfer incentives in Uganda. For the prospective enrolment, people diagnosed with TB were identified from the unit register and sought for from the waiting area and inpatient wards. Study participants were enrolled consecutively until the desired sample size was achieved for each study arm. After screening for eligibility, the study questionnaire was administered. Thereafter, simple randomization techniques were employed to allocate participants to either the intervention (Dollar arm) or routine care (Routine arm) in a ratio of 1:1. A participant randomly picked a pre-sealed opaque study envelope with a paper inside indicating the study arm of their subsequent allocation from a stack of shuffled study envelopes. The study assistant opened the envelope and revealed the allocation arm therein to the participant. At the start of the study, the stack of study envelopes had 60 envelopes with papers denoting the “Dollar arm” and 60 envelopes with papers indicating the “Routine arm” inside the envelopes. The stack was shuffled at the start of each randomization process for each patient. A second control group (Retrospective controls) comprised of participants that had a treatment outcome documented in the 6 months preceding the trial who were consecutively sampled from the unit register, starting with October 2018 backwards. The retrospective controls were intended to account for possible temporal change in treatment outcomes during the trial period considering that the intervention was administered by the health workers. In summary, the study population consisted of two groups, that is, an intervention arm (Dollar arm) and a Control group (comprised of both the Retrospective controls and the Routine arm).

### The study intervention

Immediately after randomisation (at month 0), participants allocated to the Dollar arm received 4000 Uganda shillings cash incentive, equivalent to one United States dollar (USD). They were informed that the same amount will be disbursed to them at the subsequent routine visits. Also, the participant was informed that they were at liberty to spend the incentive as they wished. Therefore, the incentive was only conditioned on turning up for the clinic visit. Subsequently, participants in the Dollar arm received the same amount of money in cash at each of their routine clinic visits at months 2, 5, and 6. During routine clinic visits, sputum samples were collected and drug refills were provided by the hospital staff as recommended by the Uganda national guidelines^18^. The cash was disbursed by the TB drug dispenser at the TB unit and participants acknowledged receipt of the cash by signing study payment vouchers. All other treatments were provided according to the standard of care by the usual care team at the hospital. Participants in the Routine arm received the usual standard of care but did not receive any money or other incentives. Health workers at the study site routinely follow up people who fail to keep clinic appointments. The study team was not involved in patient follow up. The study was registered with the Pan African Clinical Trial Registry (Registration: PACTR201908538333264): https://pactr.samrc.ac.za/TrialDisplay.aspx?TrialID=6055, date: 14/08/2019.

### Study measurements

A study questionnaire was administered to collect data on socio-demographic data, medical history, HIV status and TB related characteristics from study participants in the prospective arms. The participant’s socio-economic position at baseline was assessed using the number of durable household items possessed using the equity tool^19^ as is commonly done in national demographic surveys in Uganda^20^. Participants residing outside municipalities, town councils and town boards of their respective districts were classified to be rural residents according to the Uganda Bureau of Statistics^17^. For the retrospective controls, only socio-demographic and clinical characteristics of participants were abstracted from the unit TB register using a pre-tested data abstraction form.

Participants with TB were diagnosed and managed according to the Uganda national TB treatment guidelines by the healthcare workers at the facility. Accordingly, TB was bacteriologically confirmed by positive sputum smear microscopy and/or the Xpert MTB/Rif assay, both performed according to standard national guidelines^21^. Sputum mycobacterial load was graded as low (Xpert MTB/Rif cycle threshold (Ct) value of >22), medium (Ct values of 16–22 or smear grade of 2+) and high (Ct value > 16 or smear grade of 3+). Participants were clinically diagnosed with TB when there was no evidence of bacteriological confirmation, but the attending clinician decided to initiate a full course of TB treatment following clinical history and chest radiography. Participants were initiated on a 6-months’ regimen comprising of a two-month intensive phase of rifampicin, isoniazid, ethambutol and pyrazinamide and a 4-month continuation phase of rifampicin and isoniazid^18^. The medicines were given as fixed drug combinations under a community-based therapy model. TB treatment outcomes, extracted from the unit TB register, were determined at month eight from the time of treatment initiation of the last enrolled participant. The study end points were the TB treatment outcomes: TB cure, treatment completion, loss to follow-up and death in accordance to WHO definitions^22^.

### Blinding

Pre-sealed study envelops were delivered to the facility at the start of the study. Participant randomisation and extraction of retrospective data was conducted by a study assistant who was not part of the participants’ care team. Although the study participants and TB drug dispenser were aware of study allocation, participants were asked not to reveal their group during documentation of the treatment outcome in the TB unit register. Further, TB treatment outcomes were documented in the TB register by the unit in-charge and the district tuberculosis and leprosy supervisor who were blind to participant study allocation. Extraction of treatment outcomes from the register was conducted by a research assistant who was blind to the participant allocation.

### Sample size estimation

To determine the association of a one-dollar incentive on treatment success of people with TB, we considered a 14.9% increase in the TB success rate as observed in a cash incentive study among people with TB in rural Nigeria^23^. We also assumed a baseline TB treatment success rate of 66.7% as observed in a rural setting in Uganda^5^. Therefore, for a study power of 80%, and a two-sided α of 5% for proportion outcomes, the sample size was estimated as 135 participants per prospective arm using an online sample size calculator: OpenEpi Version 3^24^. Similarly, assuming a baseline loss to follow-up rate of 16.6%^5^ in rural Uganda and a reduction of 15.2%^23^ in loss to follow-up as a result of the intervention, the adequate sample size to determine the effect of the cash incentive on loss to follow-up would be 55 participants per prospective arm. Therefore, considering the larger of the two sample size estimations, a total of 270 participants would have been appropriate for the prospective enrolment. A second control group of 135 participants from a retrospective cohort was also considered. Therefore, the analysis was intended to involve 135 in the dollar arm, 135 in the routine arm and 135 retrospective controls. The sample size was to be distributed between the two proposed sites: 180 at MRRH and 225 at Nyeri Provincial General Hospital proportional to the average annual number of people with TB initiating treatment at the facilities. However, due to a shortfall in funding, participants were only enrolled at MRRH. As such, for the results presented, the study power to detect significant difference in cure, loss-to-follow-up and overall treatment success is 45%, 44% and 29% respectively.

### Data management and analysis

Data were entered in EpiData version 3.1 and exported to Stata 14 (StataCorp, College Station, TX, USA) for analysis. Univariate continuous variables were summarised as median and corresponding interquartile range while categorical data were summarised as frequencies. Using principal component analysis, study participants were classified into three groups: poor, middle class and rich. Baseline characteristics were compared between the Dollar arm and Control group using Pearson’s chi-square test and Fisher’s exact test where applicable. We primarily employed an intention-to-treat analysis approach. However, a sub-analysis comparing participants who received all four incentives with the control group and those who received fewer than four incentives was also performed. Treatment success was calculated as the sum of TB cure and TB treatment completion. To determine the association of the intervention on treatment outcomes, the treatment outcomes were first compared between the Dollar arm and the Control group using Pearson’s chi-square and Fisher’s exact test as appropriate. We then used multivariable Poisson regression models with robust variances that controlled for residence (rural vs urban) and history of previous TB treatment to determine the association of the intervention on the overall treatment success, cure, death, and loss to follow-up. For each model, variables that had a threshold *p *value ≤ 0.2 at bivariate analysis and/or changed estimates by at least 10% were also included in the multivariate analyses. Factors associated with the study outcomes were established at statistical significance (*p* < 0.05) and 95% confidence interval (CI). We performed complete case analysis for the Poisson regression models.

### Ethical approval and consent

Study participants in the prospective enrolment provided written informed consent and were at liberty to withdraw from the study without consequence to their regular TB care. Approval to conduct the study was obtained from The AIDS Support Organisation (TASO) research and ethics committee (Ref No. TASOREC/044/18-UG-REC-009), Uganda National Council for Science and Technology (Ref No. HS254ES), Mount Kenya University Ethics Review Committee (Ref No. MKU/ERC/0717) and Kenya national commission for science, technology and innovation (Ref No: NACOSTI/P/19/87064/27284). Waiver of consent for retrospective data was provided by TASO research and ethics committee and Mount Kenya University Ethics Review Committee. All methods and experiments were conducted according to the Declaration of Helsinki.

## Results

### Participant enrolment

**Figure 1:**
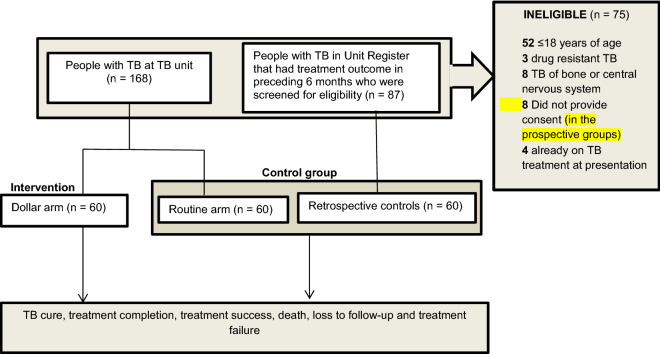
Study flow diagram.

Overall, 255 potentially eligible participants were assessed, 75 (29.4%) were ineligible, of whom 52 (69.3%) were below 18 years of age. A total of 180 participants (120 controls and 60 in the intervention arm) were enrolled and followed up between November 2018 and October 2019, the last month of study follow up period. The study flow diagram is shown in Fig. 1.

### Characteristics of study participants

Of 180 participants, 99 (55.0%) were males, 122 (67.8%) were rural residents and 77 (44.3%) were HIV co-infected. The median (interquartile range) age was 38 (28–50) years and TB was bacteriologically confirmed among 87 (48.3%) participants. Mycobacterial load grading was done for 62 (71.3%) and was low, medium and high among 19 (30.6%), 23 (37.1%) and 20 (32.3%) people, respectively. There were no significant differences in baseline characteristics between the Dollar arm and the Control group as shown in Table 1. However, sub-group analyses revealed that there were more participants in the Routine arm with history of previous TB treatment than in the Dollar arm (26.7% vs. 11.7%, p =0.037) and the Retrospective controls (26.7% vs. 8.3%, p = 0.008). There were also more rural residents in the Routine arm than the Retrospective controls (81.4% vs. 55.0%, p =0.002). There were no other statistically significant differences in the baseline characteristics among participants in the Dollar arm, Routine arm or Retrospective controls as shown in Table 2. There were missing variables for the retrospective controls since these are not provided for in the TB register.

**Table 1:** Baseline characteristics of study participants.

Characteristic	Dollar arm (N = 60)	Control group (N = 120)	*p* value
**Sex**			0.841
Male	34 (56.7)	65 (55.1)	
Female	26 (43.3)	53 (44.9)	
**Age**^**†**^			0.526
≤ 38	34 (56.7)	62 (51.7)	
> 38	26 (43.3)	58 (48.3)	
**Residence**			0.971
Rural	41 (68.3)	81 (68.1)	
Urban	19 (31.7)	38 (31.9)	
**Tuberculosis (TB) class**			0.817*
Pulmonary bacteriologically confirmed TB	28 (46.7)	59 (49.2)	
Pulmonary clinically diagnosed TB	29 (48.3)	57 (47.5)	
Extra pulmonary TB	3 (5.0)	4 (3.3)	
**HIV status at enrolment**^**†**^			0.199
Negative	34 (63.0)	63 (52.5)	
Positive	20 (37.0)	57 (47.5)	
ART status at enrolment*			0.177
On ART	14 (70.0)	30 (52.6)	
Not on ART	6 (30.0)	27 (47.4)	
**History of TB**			0.309
Yes	7 (11.7)	21 (17.5)	
None	53 (88.3)	99 (82.5)	
**Patient type at enrolment**^**‡**^			0.810
New	53 (88.3)	101 (87.1)	
Relapse	7(11.7)	15 (12.9)	
**Mycobacterial sputum grade (n = 62)**			0.192
Low	8 (26.7)	11 (34.4)	
Medium	9 (30.0)	14 (43.8)	
High	13 (43.3)	7 (21.9)	

*n = 20 for Dollar arm and n = 57 Control group.

^†^For 6 participants in the Dollar arm, the HIV status was unknown, ART—antiretroviral therapy, PTB—pulmonary tuberculosis.

^‡^For 7 participants with the Control group, the patient type was unknown.

^†^Age was categorised according to the median age of the participants.

**Table 2:** Comparison of baseline characteristics of participants in the study groups.

Characteristic	Dollar arm (N = 60) n (%)	Routine arm (N = 60) n (%)	Retrospective controls (N = 60) n (%)	*p *value^ψ^	*p* value^#^	*p *value^‡^
**Sex**				0.714	0.980	0.697
Male	34 (56.7)	32 (53.3)	33 (56.9)			
Female	26 (43.3)	28 (46.7)	25 (43.1)			
**Age**				0.714	0.464	0.715
≤ 38	34 (56.7)	32 (53.3)	30 (50.0)			
>38	26 (43.3)	28 (46.7)	30 (50.0)			
**Residence**				0.133	0.102	**0.002**
Rural	41 (68.3)	33 (55.0)	48 (81.4)			
Urban	19 (31.7)	27 (45.0)	11 (18.6)			
**TB class**				1.000^1^	0.816^1^	0.856^1^
Pulmonary bacteriologically confirmed TB	28 (46.7)	28 (46.7)	31 (51.7)			
Pulmonary clinically diagnosed TB	29 (48.3)	30 (50.0)	27 (45.0)			
Extra pulmonary TB	3 (5.0)	2 (3.3)	2 (3.3)			
**HIV status**^**†**^				0.388	0.164	0.583
Negative	34 (63.0)	33 (55.0)	30 (50.0)			
Positive	20 (37.0)	27 (45.0)	30 (50.0)			
ART status*				0.314	0.160	0.675
On ART	14 (70.0)	15 (55.6)	15 (50.0)			
Not on ART	6 (30.0)	12 (44.4)	15 (50.0)			
**History of TB**				**0.037**^**1**^	0.543	**0.008**
Yes	7 (11.7)	16 (26.7)	5 (8.3)			
None	53 (88.3)	44 (73.3)	55 (91.7)			
Cotrimoxazole use*				1.000^1^		
Yes	20 (100.0)	27 (100.0)	–			
No	0 (0.0)	0 (0.0)	–			
**Previous TB episode**^**§**^				0.525^1^		
Once	7 (100.0)	13 (81.3)	–			
Twice	0 (0.0)	3 (18.8)	–			
**Education level**				0.811^1^		
None	17 (28.3)	16 (26.7)	–			
Primary	27 (45.0)	29 (48.3)	–			
Secondary	9 (15.0)	11 (18.3)	–			
Tertiary	7 (11.7)	4 (6.7)	–			
**History of smoking**				0.111		
Ever	22 (36.7)	14 (23.3)	–			
Never	38 (63.3)	46 (76.7)	–			
**Alcohol use**				0.449		
Ever	40 (66.7)	36 (60.0)	–			
Never	20 (33.3)	24 (40.0)	–			
**Employment Status**				0.353		
Employed	33 (55.0)	38 (63.3)	–			
Unemployed	27 (45.0)	22 (36.7)	–			
**Housing status**				0.198		
Renting	30 (50.0)	23 (38.3)	–			
Own house	30 (50.0)	37 (61.7)	–			
**Number of dependents**				0.910		
≤ 3	36 (61.0)	36 (60.0)	–			
> 3	23 (39.0)	24 (40.0)	–			
**Number of rooms in housing unit**				0.336		
1–3	42 (70.0)	37 (61.7)	–			
4–6	18 (30.0)	23 (38.3)	–			
**Wealth index**				0.122		
Rich	25 (41.7)	15 (25.0)	–			
Middle	19 (31.7)	21 (35.0)	–			
Poor	16 (26.6)	24 (40.0)	–			
**Admission status at enrolment**				1.000		
Inpatient	27 (45.0)	27 (45.0)	–			
Outpatient	33 (55.0)	33 (55.0)	–			

^†^For 6 participants in the Dollar arm, the HIV status was unknown.

^§^Percentages are for participants with history of TB treatment who had data available.

ART—antiretroviral therapy, TB—tuberculosis, “–” denotes missing variable.

^1^Fisher’s exact test, *n = 27 for Routine arm, n = 20 for Dollar arm and n = 30 for Retrospective controls.

^ψ^*p* value compares Dollar arm and Routine arm.

^#^*p *value compares Dollar arm with Retrospective controls.

^‡^*p *value compares Routine arm with Retrospective controls.

### Association of one-dollar incentive with TB treatment outcomes

The overall (n = 180) treatment success rate was 62.8%; 37.8% completed treatment, 25.0% were cured, 17.2% were lost to follow-up, 17.8% died and 2.2% experienced treatment failure. In the Dollar arm, 27 (45%) received all the four incentives while 18 (30.0%) received the incentive only one. The overall treatment success rate was higher in the Dollar arm (70.0%) than the control group (59.2%), *p* = 0.156. The TB cure rate was also higher in the Dollar arm than the Control group while treatment loss-to-follow-up was lower in the Dollar arm, although this was not statistically significant, as shown in Table 3. Treatment failure was observed only in the Control groups, that is, 2 (3.3%) in the Routine arm and 2 (3.3%) in the Retrospective controls. Treatment outcomes were similar in the Control groups (Supplementary Table S1).

**Table 3:** Treatment outcomes in the dollar arm and control group.

Outcomes	Dollar arm (N = 60)	Control group (N = 120)	*p* value^1^
**Treatment completion**			0.828
No	38 (63.3)	74 (61.7)	
Yes	22 (36.7)	46 (38.3)	
**Cured**			0.068
No	40 (66.7)	95 (79.2)	
Yes	20 (33.3)	25 (20.8)	
**Lost to follow up**			0.070
No	54 (90.0)	95 (79.2)	
Yes	6 (10.0)	25 (20.8)	
**Death**			0.581
No	48 (80.0)	100 (83.3)	
Yes	12 (20.0)	20 (16.7)	
**Treatment failure**			0.303
No	60 (100.0)	116 (96.7)	
Yes	0	4 (3.3)	

In the adjusted models for factors associated with TB cure and loss-to- follow-up, participants in the Dollar arm were more likely to be cured (adjusted incidence rate ratio (aIRR): 1.59, 95% CI 1.04–2.44, *p* = 0.032) and less likely to be lost to follow-up (aIRR: 0.44, 95% CI 0.20–0.96, *p* = 0.040). History of previous TB treatment, mycobacterial grade and wealth index were not associated with any of the treatment outcomes at bivariate analysis. Table 4 shows Poisson regression analysis for factors associated with cure, loss to follow-up and treatment success.

**Table 4:** Multivariable Poisson regression models for factors associated with TB cure, loss to follow-up and treatment success.

Characteristic	Unadjusted incidence rate ratio (95% CI)	*p *value	Adjusted incidence rate ratio (95% CI)	*p *value
**Factors associated with cure**				
*Treatment allocation*				
Control group	1		1	
Dollar arm	1.60 (0.97–2.64)	0.066	1.59 (1.04–2.44)	**0.032**
*Tuberculosis class*				
Pulmonary bacteriologically confirmed TB	1		1	
Pulmonary clinically diagnosed TB	0.02 (0.00–0.17)	< 0.001	0.03 (0.00–0.19)	**< 0.001**
Extra pulmonary TB	0.29 (0.05–1.81)	0.184	0.31 (0.04–2.09)	0.227
*HIV status*				
Negative	1		1	
Positive	0.61 (0.35–1.07)	0.084	0.87 (0.53–1.44)	0.597
*Residence*				
Rural	1		1	
Urban	0.90 (0.51–1.58)	0.709	0.88 (0.54–1.44)	0.612
*Previous TB treatment*				
No	1		1	
Yes	0.84 (0.39–1.79)	0.643	0.88 (0.45–1.71)	0.698
**Factors associated with loss to follow up**				
*Treatment allocation*				
Control group	1		1	
Dollar arm	0.48 (0.21–1.11)	0.086	0.44 (0.20–0.96)	**0.040**
*Sex*				
Male	1		1	
Female	0.51 (0.25–1.05)	0.069	0.46 (0.23–0.93)	**0.030**
*Tuberculosis class*				
Pulmonary bacteriology confirmed TB	1		1	
Pulmonary clinically diagnosed TB	2.47 (1.21–5.07)	0.013	2.42 (1.22–4.83)	**0.012**
Extra pulmonary TB	(empty)			
*Residence*				
Rural	1		1	
Urban	1.18 (0.60–2.29)	0.632	1.02 (0.41–2.52)	0.969
*Patient type*				
New	1		1	
Relapse	0.52 (0.13–2.01)	0.341	0.32 (0.06–1.71)	0.182
*Age (years)*				
≤38	1		1	
>38	1.07 (0.56–2.04)	0.833	0.83 (0.40–1.87)	0.969
*Previous TB treatment*				
No	1		1	
Yes	0.80 (0.30–2.13)	0.661	1.47 (0.43–5.05)	0.544
**Factors associated with treatment success**				
*Treatment groups*				
Control	1		1	
Dollar arm	1.18 (0.95–1.48)	0.140	1.15 (0.92–1.43)	0.224
*Sex*				
Male	1		1	
Female	1.12 (0.90–1.41)	0.301	1.18 (0.95–1.48)	0.141
*Residence*				
Rural	1		1	
Urban	1.01 (0.80–1.29)	0.911	0.98 (0.77–1.25)	0.869
*Tuberculosis class*				
Pulmonary bacteriologically confirmed TB	1		1	
Pulmonary clinically diagnosed TB	0.63 (0.49–0.81)	< 0.001	0.62 (0.47–0.81)	**0.001**
Extra pulmonary TB	1.13 (0.82–1.56)	0.463	1.21 (0.83–1.75)	0.325
*HIV status*				
Positive	1		1	
Negative	1.15 (0.91–1.47)	0.243	1.16 (0.90–1.51)	0.246
*Previous TB treatment*				
No	1		1	
Yes	1.17 (0.89–1.52)	0.256	1.27 (0.97–1.66)	0.085

### Sub-analysis for treatment success among participants in the Dollar arm who received all four incentives

The treatment success rate among participants in the dollar arm who received all four incentives (n = 27) was 96.3%. Of these, 12 (44.4%) were cured, 14 (51.9%) completed treatment and only one (3.7%) participant died. The treatment success rate was significantly higher among participants who received all the four incentives than all other sub-groups (Table 5). Receiving all four incentives was associated with treatment success (aIRR: 1.61, 95% CI 1.35–1.91, *p* < 0.001) in a multivariable model that adjusted for number of incentives, rural residence, and history of TB treatment (Supplementary Table S2).

**Table 5:** Comparison of treatment success between participants who received all four incentives and other sub-groups.

Treatment group	Treatment success n (%)	No treatment success n (%)	*p *value^**†**^
**Comparison with entire control group**			< 0.001
Received 4 incentives (n = 27)	26 (96.3)	1 (3.7)	
Control group (n = 120)	71 (59.2)	49 (40.8)	
**Comparison with retrospective controls**			< 0.001
Received 4 incentives (n = 27)	26 (96.3)	1 (3.7)	
Retrospective controls (n = 60)	33 (55.0)	27 (45.0)	
**Comparison with routine arm**			0.001
Received 4 incentives (n = 27)	26 (96.3)	1 (3.7)	
Prospective controls (n = 60)	38 (63.3)	22 (36.7)	
**Comparison within Dollar arm**			< 0.001
Received 4 incentives (n = 27)	26 (96.3)	1 (3.7)	
Received 1 to 3 incentives (n = 33)	16 (48.5)	17(51.5)	
**Comparison with all other categories**			< 0.001
Received 4 incentives (n = 27)	26 (96.3)	1 (3.7)	
Received 0–3 incentives*	87 (56.9)	66 (43.1)	

*Includes control group and participants who received < 4 incentives in the dollar arm.

^**†**^Fisher’s exact test.

## Discussion

We evaluated the association of a one-dollar incentive with TB treatment outcomes at a rural hospital in Uganda using a quasi-experiment consisting of a two-arm pragmatic randomised controlled trial and an additional control group from a retrospective cohort. The study was under-powered due to failure to achieve the desired sample size. Nevertheless, we found that receiving the incentive was associated with 59% increase in the rate of TB cure and a 56% reduction in treatment loss-to-follow-up compared to not receiving the incentive. Further, over-all treatment success was higher among participants who received the incentive by 11 percentage points, although this did not reach statistical significance, probably due to a low study power. Additionally, all cases of treatment failure were observed in the Control group. Moreover, among participants who received all four incentives the treatment success rate was more than the 90% global target.

It is apparent that a small cash incentive has the potential to improve TB treatment outcomes in rural settings and should be further explored by larger studies. Such a modest incentive may be scalable to routine programmatic settings in low and middle-income settings (LMICs) where the TB treatment success is low^2^. Conditioned cash incentives foster patients’ adherence to clinic appointments and treatments which ultimately results in a favourable outcome^25^. In TB treatment, adherence to clinic appointments and treatments would ensure that patients complete the treatment schedule as prescribed and monitoring of sputum smear positivity can be performed to ascertain the treatment outcome as “treatment completion” or “cure”^22^. One Cochrane review found that higher cash incentives are more effective than lower cash incentives in promoting TB prophylaxis treatment adherence^13^. Several studies have shown that cash incentives improve TB treatment outcomes in LMICs although they involve a large sum of money which TB high-burdened countries cannot afford^23,26–29^. Additionally, most of the studies are before-and-after studies or purely historical cohorts^23,27,28,30,31^. It is therefore difficult to make straightforward comparisons between these study designs and our current study.

Similar to our findings, Ukwanja et al. found cash incentives to significantly improve TB treatment outcomes when they compared a retrospective cohort of people with TB with a prospective group that received a cash incentive in rural Nigeria^23^. In their study, a 75% reduction in loss-to-follow-up was observed in the intervention period. However, they offered a significantly higher incentive (15 USD) per month and the comparison with a historical cohort does not take into account temporal changes in treatment outcomes. Similarly, a randomised controlled trial in Peru found that a cash incentive coupled with community meetings and household visits significantly increased treatment success and cure^26^. It was, however, difficult to attribute the improvement to the cash incentive alone, the households were the unit of randomisation and a large amount of the incentive (230 USD per household) was involved as well. These factors may explain why the effect of the incentive in our study was significant with regards to cure and loss to follow-up but not over- all treatment success as observed in these studies. Our study population was also predominantly clinically diagnosed and these individuals were likely to be lost to follow-up possibly because they had an alternative diagnosis or died. To increase treatment success in rural settings, the diagnostic capabilities of rural facilities need to be improved to enable bacteriological confirmation of TB. Similar to our finding, a hospital-based study in rural Papua New Guinea also reported a high proportion of participants with clinically diagnosed TB who had a higher frequency of unsuccessful outcomes^32^. Interestingly, despite having more participants with clinically diagnosed TB than the Control group, the treatment success in the Dollar arm was similar to that reported (70.5%) among bacteriologically confirmed People with TB in rural Uganda^33^.

Contrary to our findings, in South India, a recent study found that a monthly 8 USD incentive did not improve treatment outcomes among TB/HIV co-infected participants^31^. However, this study also involved a before-and-after analysis and in 20% and 25% of participants the incentive was not disbursed and delayed respectively.

We observed lower rate of loss-to-follow-up among females. This is similar to the association of treatment success with female gender reported in rural Nigeria^23^. Compared to females, males in rural Uganda are more likely to be lost to follow-up or die during TB treatment^33^. It is unknown whether cash incentives have different effects on the treatment outcomes among males and females. This would be an area for further evaluation considering that the two sexes experience similar TB catastrophic costs^34^.

Our study had limitations. The sample size was small because we were unable to complete participant enrolment at the second site. The study was therefore not well powered, and this could have affected the estimate of the true effect of the incentive. Therefore, we recommend a larger study to be conducted in rural programmatic settings. The multiple sub-category comparisons potentially increase the risk for a chance occurrence of statistical significance. The multivariable analyses performed attempt to obviate this limitation. Additionally, our study is from a single-centre regional referral hospital. While this may affect the generalizability of our findings, it is evident that studies from similar settings have reported similar results. For the retrospective controls, several variables could not be assessed since they are not routinely documented in the TB register. Therefore, we may not have adjusted for some potential confounders. Nonetheless, the variables we assessed are similar to those from previous studies^23,26,31^. We did not assess for treatment adherence which influences TB cure. However, keeping of clinic appointments and loss-to-follow-up are proxies of treatment adherence. Lastly, we were unable to adjust for some measures of TB severity such as cavitary disease on radiological imaging because these data were unavailable. However, we analysed for the association of other proxies of TB severity: previous TB retreatment, sputum smear grade, extrapulmonary TB, and HIV co-infection and treatment outcomes and found no association. The strength of our study lies in the design, that is, we were able to control for temporal changes in treatment outcomes by having both retrospective and prospective controls. Further, we used a small incentive under programmatic settings. If validated by larger studies, this intervention is likely to be more sustainable in low-income settings.

## Conclusion

A one-dollar incentive was associated with higher TB cure, lower loss-to-follow-up and resulted in a clinically significant improvement in treatment success among people with TB in rural Uganda. A cost-effectiveness analysis to assess the translation of such interventions into routine programmatic TB care is desirable.

## Supplementary Information

Supplementary Tables.

## Data Availability

Datasets used in this analysis are available from the corresponding author upon reasonable request.
