# Supplementary material for: One dollar incentive improves tuberculosis treatment outcomes in programmatic settings in rural Uganda
Source: Sci Rep. 2021 Sep 29;11:19346. doi: 10.1038/s41598-021-98770-7 (PMC8481464; doi:10.1038/s41598-021-98770-7)
Supplement: Supplementary file 1 — Supplementary Tables. [file 41598_2021_98770_MOESM1_ESM.docx]

Supplementary table 1: Comparison of treatment outcomes between the dollar arm and the individual control groups

| Outcomes | Dollar arm | Routine arm | Retrospective arm | p-value^1^ | p-value^2^ |
| --- | --- | --- | --- | --- | --- |
| Treatment completion |  |  |  | 0.707 | 1.000 |
| No | 38 (63.3) | 36 (59.3) | 38 (63.3) |  |  |
| Yes | 22 (36.7) | 24 (40.7) | 22 (36.7) |  |  |
| Cured |  |  |  | 0.224 | 0.061 |
| No | 40 (66.7) | 46 (76.7) | 49 (81.7) |  |  |
| Yes | 20 (33.3) | 14 (23.3) | 11 (18.3) |  |  |
| Lost to follow up |  |  |  | 0.283 | **0.031** |
| No | 54 (90.0) | 50 (83.3) | 45 (75.0) |  |  |
| Yes | 6 (10.0) | 10 (16.7) | 15 (25.0) |  |  |
| Death |  |  |  | 0.637 | 0.637 |
| No | 48 (80.0) | 50 (83.3) | 50 (83.3) |  |  |
| Yes | 12 (20.0) | 10 (16.7) | 10 (16.7) |  |  |
| Treatment failure |  |  |  | 0.496* | 0.496* |
| No | 60 (100.0) | 58 (96.7) | 58 (96.7) |  |  |
| Yes | 0 | 2 (3.3) | 2 (3.3) |  |  |

^1^p-value compares Dollar arm to Routine arm, ^2^p-value compares Dollar arm to retrospective arm, *Fisher’s exact test.

Supplementary table 2: Factors associated with treatment success (adjusted for number of incentives received)

| Characteristic | Unadjusted  Incidence rate ratio (95%CI) | p-value | Adjusted  Incidence rate ratio (95%CI) | p-value |
| --- | --- | --- | --- | --- |
| Number of incentives received |  |  |  |  |
| 0-3 incentives* | 1 |  | 1 |  |
| 4 incentives | 1.69 (1.45 – 1.98) | <0.001 | 1.61 (1.35 – 1.91) | **<0.001** |
| Sex |  |  |  |  |
| Male | 1 |  | 1 |  |
| Female | 1.13 (0.90 – 1.41) | 0.301 | 1.14 (0.91 – 1.42) | 0.245 |
| HIV status |  |  |  |  |
| Positive | 1 |  | 1 |  |
| Negative | 1.15 (0.91 – 1.47) |  | 1.17 (0.91 – 1.41) | 0.233 |
| Tuberculosis class |  |  |  |  |
| Pulmonary bacteriologically confirmed | 1 |  | 1 |  |
| Pulmonary clinically diagnosed | 0.63 (0.49 – 0.81) | <0.001 | 0.64 (0.49 – 0.83) | **0.001** |
| Extra pulmonary TB | 1.13 (0.82 – 1.56) | 0.463 | 1.25 (0.85 – 1.84) | 0.255 |
| Residence |  |  |  |  |
| Rural | 1 |  | 1 |  |
| Urban | 1.01 (0.80 – 1.29) | 0.911 | 1.02 (0.80 – 1.30) | 0.851 |
| History of previous TB |  |  |  |  |
| No | 1 |  | 1 |  |
| Yes | 1.17 (0.89 – 1.51) |  | 1.25 (0.96 – 1.62) | 0.097 |

*includes control group and participants who received < 4 incentives in the dollar arm
